# Protection of Corneal Limbus from Riboflavin Prevents Epithelial Stem Cell Loss after Collagen Cross-Linking

**DOI:** 10.1155/2018/6854298

**Published:** 2018-06-03

**Authors:** Hyo Kyung Lee, Jin Suk Ryu, Hyun Jeong Jeong, Mee Kum Kim, Joo Youn Oh

**Affiliations:** ^1^Department of Ophthalmology, Seoul National University Hospital, 101 Daehak-ro, Jongno-gu, Seoul 110-744, Republic of Korea; ^2^Laboratory of Ocular Regenerative Medicine and Immunology, Biomedical Research Institute, Seoul National University Hospital, 101 Daehak-ro, Jongno-gu, Seoul 110-744, Republic of Korea

## Abstract

**Purpose:**

To investigate whether the protection of corneal limbus from riboflavin exposure during collagen cross-linking (CXL) prevents limbal epithelial stem cell (LESC) loss.

**Methods:**

Ten New Zealand white rabbits received an epithelium-off CXL using an accelerated protocol. Seven days before procedure, 5-bromo-2-deoxyuridine (BrdU) was intraperitoneally injected. During procedure, riboflavin was applied to the corneal surface within a 9 mm diameter retention ring in 5 rabbits, thereby preventing the limbus from riboflavin exposure. In other 5 rabbits, riboflavin was instilled every 2 min, allowing the spillover to the limbus. One day after UVA irradiation, corneas were subjected to histological and molecular assays.

**Results:**

There were no differences in corneal thickness and epithelial healing between the groups. The numbers of BrdU-labelled and p63^+^ limbal epithelial cells were markedly reduced in the group without a ring, but significantly increased when a ring was used. Robust expression of CK3/12 was observed in the limbal epithelium in the group with a ring. The mRNA levels of ABCG2, FGF2, IL-1*β*, and IL-6 were significantly increased in the corneas with a ring.

**Conclusions:**

Protection of limbus from riboflavin during CXL was effective in preserving LESCs. However, inflammation was increased in the cornea treated with riboflavin using a ring.

## 1. Introduction

Corneal ectatic disorders including keratoconus and postoperative keratectasia are one of the most common causes leading to vision loss in young adult population [1, 2]. For the treatment of corneal ectatic disorders, corneal collagen cross-linking (CXL) has recently emerged as a promising tool to halt ectasia progression and cause regression [2].

The CXL procedure involves topical application of riboflavin to the corneal surface and ultraviolet-A (UVA) irradiation. The reactive oxygen species (ROS) generated during UVA irradiation induces covalent bonds in corneal collagen fibrils. As a result, corneal biomechanical stability is increased and the ectatic corneal tissue strengthened. However, both UVA-induced ROS and UVA itself can cause DNA damage in a cell, leading to cell death [3].

In this regard, several reports previously demonstrated the cytotoxic effects of riboflavin-UVA on corneal cells including limbal epithelial stem cells (LESCs) [4–12]. Also, there are case reports of delayed corneal epithelial healing in patients after CXL [9, 13, 14]. It was reported that covering the limbus with a metal or PMMA shield during UVA irradiation preserved LESCs in cultures of human corneal epithelial cells or in human cadaver eyes [10, 11, 15]. However, to better identify adverse effects of CXL on LESCs and to evaluate beneficial effects of limbal protection on the cells, an in vivo study is essential because an in vivo limbal microenvironment is important for LESC survival, and cell apoptosis can be influenced by systemic immune system, not only by direct toxic effects of an injury on cells.

Therefore, we here investigated the in vivo effects of CXL on the survival of LESCs, apoptosis of corneal epithelial cells, and corneal inflammation in rabbits. In addition, since riboflavin increases UVA absorption into the cornea as a photosensitizer, we tested whether the protection of corneal limbus from riboflavin exposure during CXL might prevent the potential toxicity of UVA on LESCs [5, 12].

## 2. Materials and Methods

The experimental protocol was approved by the Institutional Animal Care and Use Committee of Seoul National University Biomedical Research Institute (number 16-0103-S1A1). Animal experiments were performed in accordance with the ARVO Statement for Use of Animals in Ophthalmic Vision and Research.

### 2.1. Animals

**Figure 1:**
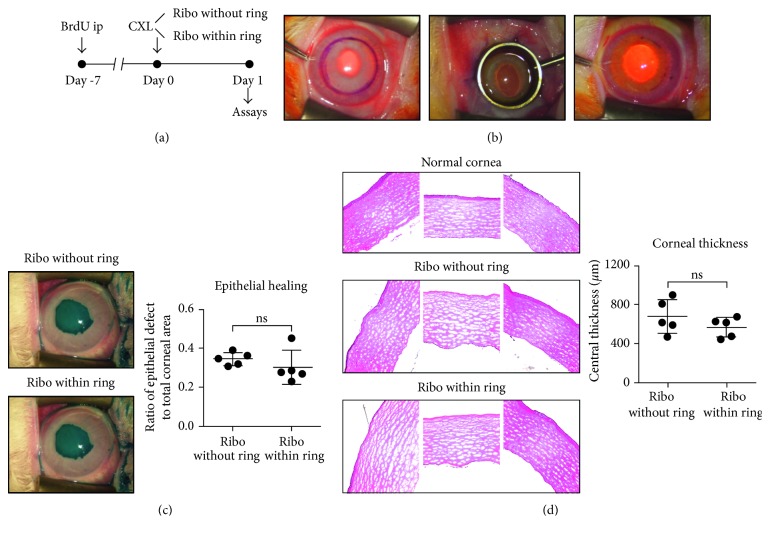
Experimental scheme and examination of corneal epithelial healing and thickness. (a) Scheme of experiments: 5-bromo-2-deoxyuridine (BrdU) was injected intraperitoneally in rabbits on Day 7, and collagen cross-linking (CXL) performed on Day 0. On Day 1, corneas were evaluated by clinical, histological, and molecular assays. During CXL, riboflavin was instilled over the corneal and limbal surface every two min in group 1 rabbits (riboflavin without ring), and group 2 rabbits received riboflavin using a retention ring to avoid the limbus (riboflavin within ring). (b) Photographs of CXL procedure in the group 2 (riboflavin within ring). After marking and scraping the central 9.0 mm corneal epithelium, a retention ring was applied and riboflavin filled within a ring. (c) On Day 1, corneal epithelial healing was examined with 3% lissamine green dye staining and quantitated by a ratio of epithelial defect area to the total corneal area. (d) Hematoxylin-eosin staining. Original magnification ×200. The central corneal thickness was measured on stained slides. Data are presented as the mean ± SD, and a dot represents a single animal. ns: not significant.

Ten New Zealand white rabbits weighing 2.1 kg to 2.5 kg (KOATECH, Gyeonggi-do, Korea) were randomly divided into two groups (groups 1 and 2; *n*=5 per group). The experimental scheme is summarized in Figure 1(a). All rabbits were treated with an intraperitoneal (IP) injection of 5-bromo-2-deoxyuridine (BrdU; Sigma, St. Louis, MO) at a dose of 50 mg/kg [16]. Seven days later, CXL was performed in the right eyes of rabbits, and the left eyes served as negative controls. One day after CXL, corneas were clinically examined and collected for histologic and molecular assays.

### 2.2. CXL Procedure

Rabbits were anesthetized with an intramuscular injection of 10 mg/kg zolazepam-tiletamine (Zoletil®, Virbac, Carros, France) and10 mg/kg xylazine hydrochloride (Rompun®, Bayer, Frankfurt, Germany). After topical instillation of 0.5% proparacaine solution (Paracaine®, Hanmi Pharm, Seoul, Korea), central 9 mm diameter corneal epithelium was marked and scraped off using a surgical blade (Figure 1(b)). After checking the central corneal thickness (CCT) which was >325 *μ*m, an isotonic 0.1% riboflavin solution (VibeX Rapid®, Avedro, Inc. Waltham, MA) was applied to the corneal surface. In group 1, a 9 mm-diameter retention ring was used for riboflavin application for 10 min (Figure 1(b)). The ring was applied to the cornea with pressure enough to prevent riboflavin leakage out of a ring, and by this means, the limbus was not exposed to riboflavin for the whole treatment period. In group 2, riboflavin drops were instilled every 2 min for 10 min, allowing the spillover of riboflavin to the limbus. After riboflavin application, the eyes were rinsed with 40 mL balanced salt solution (BSS, Alcon Laboratories, Ft. Worth, TX), and CCT was confirmed to be >325 *μ*m. Then, UVA irradiation was performed for 8 min using an Avedro system (Avedro, Inc. Waltham, MA) in a pulsed mode (30 mW/cm^2^, 1 sec on-1 sec off, a total dose of 7.2 J/cm^2^). At the end of the procedure, one drop of 0.5% levofloxacin ophthalmic solution (Cravit®, Santen, Osaka, Japan) was instilled to the cornea.

### 2.3. Measurement of CCT, Corneal Endothelial Cell Counts, and Epithelial Defects

Before and one day after the CXL procedure, CCT and corneal endothelial cell counts (ECC) were measured using an ultrasound pachymeter (Pocket II®, Quantel Medical, Bozeman, MT) and a noncontact specular biomicroscope (Konan specular microscope SP-8800, Konan Medical, Inc., Nishinomiya, Japan), respectively. Also, corneal epithelial defects were assessed with 3% lissamine green vital staining (Figure 1(c)). To quantitatively measure the size of defects, corneal photographs were taken, and the proportion of the stained area to the total corneal area was calculated using ImageJ software (US National Institutes of Health).

### 2.4. Histology

After clinical examination, rabbits were humanely killed with an intravenous injection of potassium chloride (1 mg/kg) under deep anesthesia, and corneas were extracted. The half of a cornea was subjected to histologic assays, and another half to molecular assays.

For histologic examination, the frozen sections were subjected to hematoxylin-eosin staining, immunostaining for BrdU, p63, and CK (cytokeratin) 3/12. For detection of BrdU-labelled nuclei, rat mAb to BrdU (1 : 100, ab6326, Abcam) was used. For p63 and CK 3/12 immunostaining, goat mAb to rabbit p63 (1 : 100, ab124762, Abcam), and mouse mAb to rabbit CK3/12 (1 : 100, ab68260, Abcam, Cambridge, UK) were used as primary antibodies. Secondary antibodies used were goat anti-rat IgG, TRITC (Millipore, Billerica Massachusetts 01821, USA) for BrdU and Ck3/12, and goat anti-mouse IgG Alexa 488 (Invitrogen, Waltham, MA, USA) for p63. Nuclei were counterstained using Hoechst 33342 (Sigma, St. Louis, MO, USA).

The stained slides were observed under a fluorescent microscope (BX-61, Olympus, Melville, NY, USA) with ×200 magnification. The number of positively stained cells was counted in three different sections of each eye, and the average count was determined.

### 2.5. Real-Time RT PCR

The corneal tissue was cut into small pieces by microscissors and lysed in RNA isolation reagent (RNA Bee, Tel-Test, Inc., Friendswood, TX). After sonication with a probe sonicator (Ultrasonic Processor, Cole Parmer Instruments, Vernon Hills, IL), total RNA was extracted using an RNeasy Mini kit (Qiagen, Valencia, CA), and first-strand cDNA was synthesized by reverse transcription (High Capacity RNA-to-cDNA Kit; Applied Biosystems, Carlsbad, CA). The cDNA was analyzed by real-time PCR using TaqMan Universal PCR Master Mix (Applied Biosystems) on an ABI 7500 Real-Time PCR System (Applied Biosystems) for the following molecules: rabbit ABCG2 (ATP-binding cassette sub-family G member 2), FGF2 (fibroblast growth factor 2), interleukin (IL)-1*β*, and IL-6. A rabbit GAPDH was used for normalization of gene expression. For probe sets, TaqMan Gene Expression Assay kits were purchased from Applied Biosystems. The assays were performed in triple technical replicates for each sample.

### 2.6. Statistical Analysis

GraphPad Software (GraphPad Prism®, Inc., La Jolla, CA) was used for statistical tests. To compare the means of more than two groups, data were analyzed by the Kruskal–Wallis test. Dunn's test was used for a follow-up pairwise comparison of the groups after the null hypothesis was rejected (*p* < 0.05). Comparison of values from two groups was performed with the Mann–Whitney test. Data were presented as the mean ± SD. Differences were considered significant at *p* < 0.05.

## 3. Results

### 3.1. Effects on CCT, ECC, and Corneal Epithelial Healing

The pre- and postoperative values of CCT and ECC in group 1 (riboflavin without a ring) and 2 (riboflavin within a ring) are presented in Table 1. At baseline, the CCT as measured by an ultrasound pachymeter was 384.6 ± 1.80 *μ*m in group 1 and 381.6 ± 1.60 *μ*m in group 2 (*p*=0.548 between the group 1 and 2). The ECC were 3806 ± 218.70 cells/mm^2^ and 3765 ± 110.50 cells/mm^2^ in groups 1 and 2, respectively (*p*=0.917). One day after CXL, the CCT highly increased in both groups without significant difference between two groups (*p*=0.691). Consistent with pachymetry results, hematoxylin-eosin staining showed marked stromal edema in the cornea after CXL, and the CCT as measured in hematoxylin-eosin-stained sections was not different between two groups (Figure 1(d)).

Similarly, no significant difference was observed in corneal epithelial healing between group 1 and 2 as assessed by lissamine green staining (*p*=0.151) (Figure 1(c)).

### 3.2. Effects on LESC Survival

**Figure 2:**
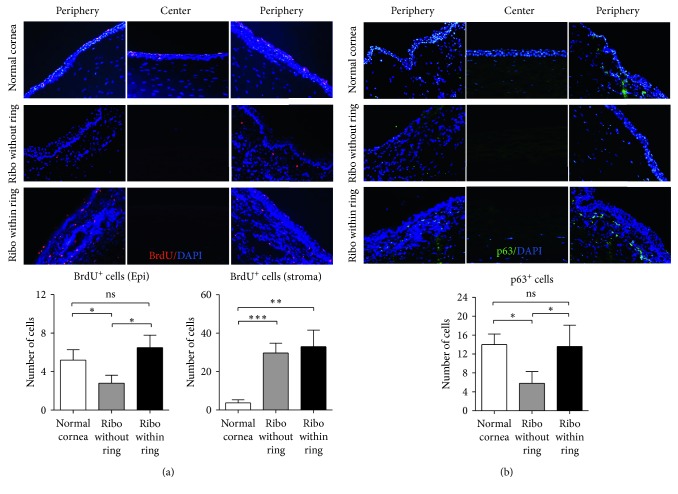
Analysis of limbal epithelial stem cells. (a) Representative images and quantitative analysis of the cells labelled with 5-bromo-2-deoxyuridine (BrdU) in the central and limbal corneal sections 8 days after pulse treatment with BrdU. The number of BrdU-labelled cells was separately counted in the epithelium (Epi) and stroma. (b) Representative images and quantitation of p63^+^ cells. Original magnification ×200. Data are presented as the mean ± SD, and a dot represents a single animal. ^*∗*^ *p* < 0.05; ^*∗∗*^ *p* < 0.01; ^*∗∗∗*^ *p* < 0.001; ns: not significant.

To assess LESCs in vivo, we resorted to two methods. First, we assayed for slowly recycling cells in the limbal epithelium as evaluated by BrdU label retaining cells 8 days after pulse treatment with IP BrdU (Figure 1(a)). BrdU-labelled cells were frequently observed (5.19 ± 3.73 per X200 section) in normal corneas, but there was a significant decrease in BrdU-labelled cells in the limbal epithelium in the group 1 corneas (riboflavin without a ring) (2.80 ± 2.61 per X200 section; *p*=0.030) (Figure 2). Notably, the number of BrdU-labelled cells in the limbal epithelium was significantly higher in the group 2 corneas (riboflavin within a ring) (6.50 ± 3.65 per X200 section) compared to the corneas in group 1 (*p*=0.021) (Figure 2(a)). Another notable finding was that BrdU-labelled cells in the stroma were significantly increased in both groups 1 and 2, while there were few BrdU^+^ cells detected in the normal corneal stroma (Figure 2(a)). Since BrdU incorporates into hematopoietic progenitor cells possessing proliferating capacity in bone marrow after pulse treatment, it is possible that BrdU-labelled cells in the corneal stroma are inflammatory cells differentiated from myeloid origin [17].

As another method for evaluating LESCs, we evaluated p63^+^ cells because p63 is known as a putative corneal epithelial stem cell marker [18]. Consistent with BrdU^+^ cells, the number of p63^+^ cells was significantly reduced in the limbus in the group 1 corneas one day after CXL (5.80 ± 2.49 per X200 section) compared to normal corneas (14.00 ± 2.24 per X200 section, *p*=0.0321). However, the number of p63^+^ cells was significantly higher in the limbus in group 2 (13.60 ± 4.51 per X200 section) than in group 1 (*p*=0.0433) and not different from normal corneas (*p* > 0.999) (Figure 2(b)).

Together, results indicate that LESCs were decreased by CXL, and protection of the limbus from riboflavin exposure prevented the loss of LESCs.

### 3.3. Effects on Corneal Epithelial Cells

**Figure 3:**
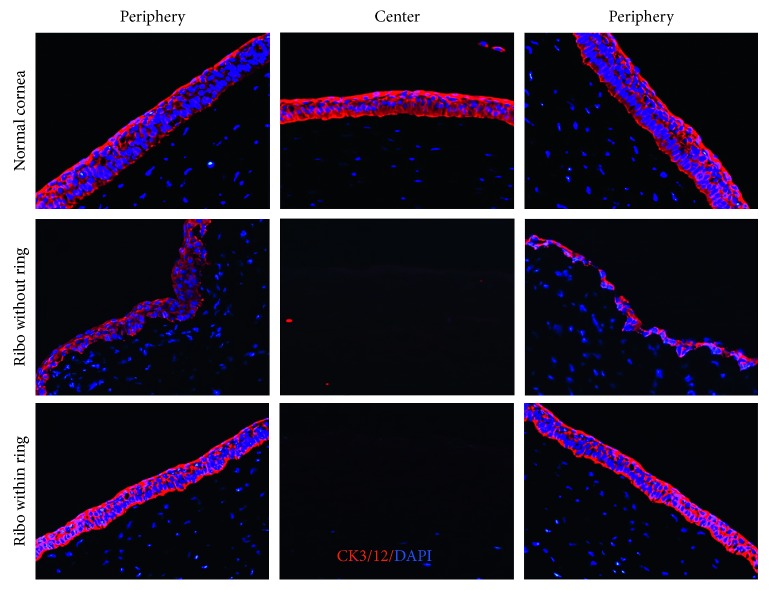
Analysis of differentiated corneal epithelial cells. Representative images of CK3/12 immunostaining in the central and limbal corneal sections. Original magnification ×200.

In addition to LESCs, we evaluated corneal epithelial cells using immunostaining for CK3/12 which is a marker for differentiated corneal epithelial cells [19]. After CXL, CK3/12 expression was markedly decreased in the limbal epithelium in the group 1 corneas (riboflavin without a ring) (Figure 3, Supplementary Figures 1 and 2). In contrast, a robust expression of CK3/12 was observed in the limbal epithelium in the group 2 corneas (riboflavin with a ring) similar to normal corneas (Figure 3, Supplementary Figure 3).

### 3.4. Effects on Stem Cell Marker and Inflammatory Cytokines

**Figure 4:**
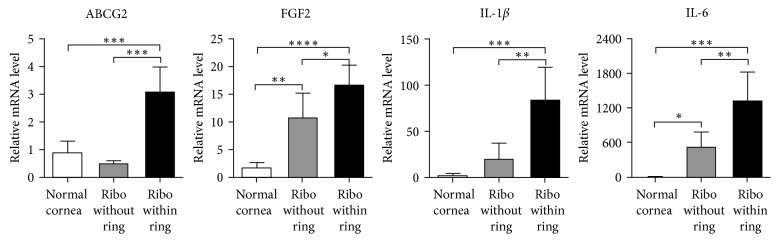
The expression of epithelial stem cell marker and inflammatory cytokines in the cornea. The mRNA levels of ABCG2 (epithelial stem cell marker) and FGF2, IL-1*β*, and IL-6 (inflammatory cytokines) as measured by real-time RT PCR. Shown were the relative values to the levels in normal corneas (mean ± SD). ^*∗*^ *p* < 0.05; ^*∗∗*^ *p* < 0.01; ^*∗∗∗*^ *p* < 0.001; ^*∗∗∗∗*^ *p* < 0.0001.

We further measured the mRNA levels of the epithelial stem cell marker (ABCG2) and proangiogenic/inflammatory cytokines (FGF2, IL-1*β*, and IL-6) in the cornea by real-time RT PCR [18]. Consistent with BrdU and p63 immunostaining (Figure 2), the level of ABCG2 was significantly decreased in the group 1 corneas (riboflavin without a ring) compared to normal controls, reflecting the loss of LESCs (Figure 4). However, there was no difference in ABCG2 levels between the group 2 (riboflavin within a ring) and normal corneas (Figure 4). The levels of FGF2, IL-1*β*, and IL-6 transcripts in corneas were significantly increased after CXL and significantly higher in group 2 than in group 1 (Figure 4).

## 4. Discussion

Our data demonstrated that CXL (riboflavin-UVA) induced the loss of LESCs and corneal epithelial cells in the limbus in rabbits. The use of a retention ring for riboflavin application and protection of the corneal limbus from riboflavin exposure protected LESCs and corneal epithelial cells against CXL-induced damage. However, inflammation was more severe in the cornea treated with riboflavin using a ring, compared to that without using a ring.

It is well-known that UVA induces apoptosis in corneal cells including LESCs, keratocytes, and corneal endothelial cells [4–7]. The UVA-induced damage is aggravated in the presence of a photosensitizer such as riboflavin. For example, it was reported that the threshold for UVA-induced damage was 5 mW/cm^2^ in keratocyte, but with riboflavin, it was lowered to the range of 0.5–0.7 mW/cm [20, 21]. Also, the UVA damage threshold in corneal endothelial cells was shown to be 10 times lower when the cells were treated with riboflavin and UVA, compared to when exposed to UVA alone [7]. Hence, limiting the exposure to riboflavin might be beneficial to protect the cells from UVA irradiation. Consistent with this hypothesis, our data showed that the protection of the limbus from riboflavin during CXL was effective in preserving LECSs and limbal corneal epithelial cells from UVA-induced damage. In line with these, other studies reported that covering the limbus with a metal or PMMA shield during CXL prevented the UVA-mediated damage to LESCs [11, 15]. Since LESCs are essential for the maintenance of a healthy corneal epithelium under both normal and wound healing conditions, efficient protection of the limbal region from riboflavin and/or UVA would help avoid the complications of CXL [22].

One interesting finding of our study was that inflammatory reaction occurred in the cornea after CXL and was more severe when a ring was used for riboflavin soaking. We did not directly measure the concentration of riboflavin, but it is likely that the riboflavin concentration was higher in the cornea where riboflavin was constantly applied within a ring, compared to the cornea where a drop of riboflavin was intermittently instilled every 2 min. This increased riboflavin and UVA reaction might, therefore, result in activation of keratocytes and immune cells in the corneal stroma, leading to enhanced inflammatory response. Alternatively, the mechanical pressure while applying a ring to the cornea for riboflavin soaking might be another cause of increased inflammation in the cornea. Regardless of the pathogenesis, inflammation causes rare but vision-threatening complications such as corneal scarring, haze, or thinning. Therefore, effective control of the postoperative inflammation would be important for achieving good outcomes after CXL.

Our study has limitations. First, we here used rabbits that have thinner CCT than humans. The CCT in rabbits used in our study was 383.12 ± 5.44 *μ*m after epithelial scraping and 358.44 ± 14.19 *μ*m after riboflavin application. It is generally recommended that UVA irradiation during CXL should be performed only when the thickness of riboflavin-saturated cornea is >400 *µ*m [5]. In fact, the marked corneal edema developed in all rabbits after CXL in our study. Also, collagen fibers are thinner in the rabbit cornea compared to the human counterpart, and the proportions of collagen type I and VI in the stroma are different between rabbit and human corneas [23, 24]. For these reasons, it is difficult to directly extrapolate our results observed in rabbits to humans. Another limitation of our study is that it was the short-term study. Therefore, further studies on the long-term results of the limbus protection during CXL on LESCs in humans would be helpful to optimize the CXL protocols for safety and efficacy.

## 5. Conclusions

In conclusion, the use of a ring as a sink to retain riboflavin was effective in protecting the limbus and LESCs during CXL. With appropriate inflammation control, it can be considered as one of useful methods in the CXL procedure.

## Figures and Tables

**Table 1:** Pre- and postoperative measurements of central corneal thickness and endothelial cell counts.

	Group 1 (*N*=5), riboflavin without ring	Group 2 (*N*=5), riboflavin within ring	*p* value
*Preoperative*			
CCT (*µ*m) (371.8–390.8)	384.6 ± 1.80 (382.8–387.6)	381.6 ± 7.60 (371.8–390.8)	0.548
ECC (cells/mm^2^) (3597–4167)	3806 ± 218.70 (3597–4167)	3765 ± 110.50 (3636–3906)	0.917

*Postoperative*			
CCT (*µ*m) (627.8–838.8)	693.5 ± 39.76 (651.4–755.0)	719.3 ± 77.55 (627.8–838.8)	0.691

CCT: central corneal thickness; ECC: endothelial cell count.

## Data Availability

All data generated or analyzed during this study are included in this published article and its supplementary files.

## Supplementary Materials

Supplementary 1 Figure 1: CK3/12 immunostaining in normal rabbit corneas. Shown were central and limbal areas.

Supplementary 2 Figure 2: CK3/12 immunostaining in the corneas treated with riboflavin not using a ring, one day after CXL.

Supplementary 3 Figure 3: CK3/12 immunostaining in the corneas treated with riboflavin using a ring, one day after CXL.
