# Supplementary figures and images for: Protection of Corneal Limbus from Riboflavin Prevents Epithelial Stem Cell Loss after Collagen Cross-Linking
Source: J Ophthalmol. 2018 Jun 3;2018:6854298. doi: 10.1155/2018/6854298 (PMC6008863; doi:10.1155/2018/6854298)

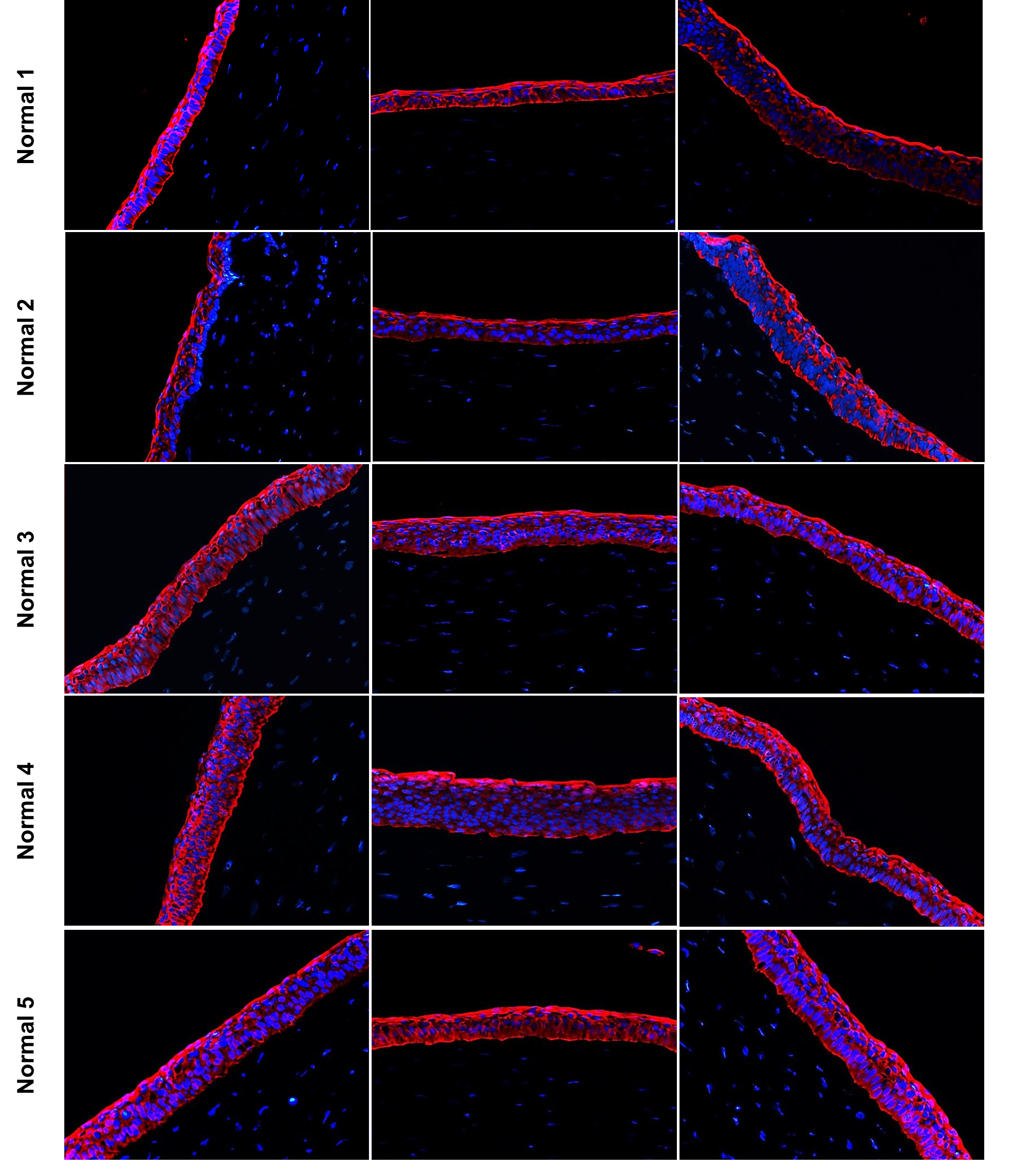

Supplement: Supplementary 1 — Figure 1: CK3/12 immunostaining in normal rabbit corneas. Shown were central and limbal areas. [file 6854298.f1.tif]

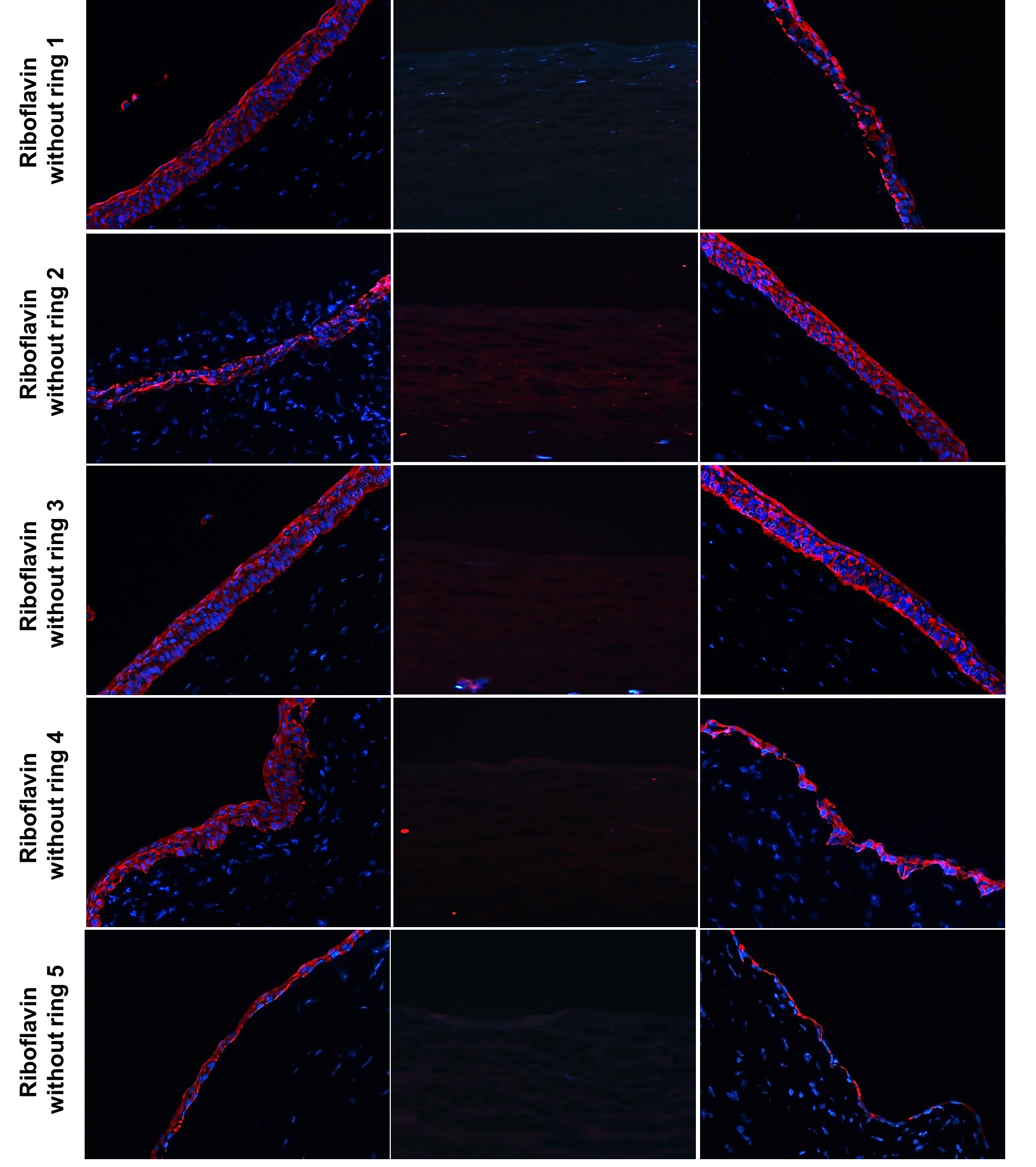

Supplement: Supplementary 2 — Figure 2: CK3/12 immunostaining in the corneas treated with riboflavin not using a ring, one day after CXL. [file 6854298.f2.tif]

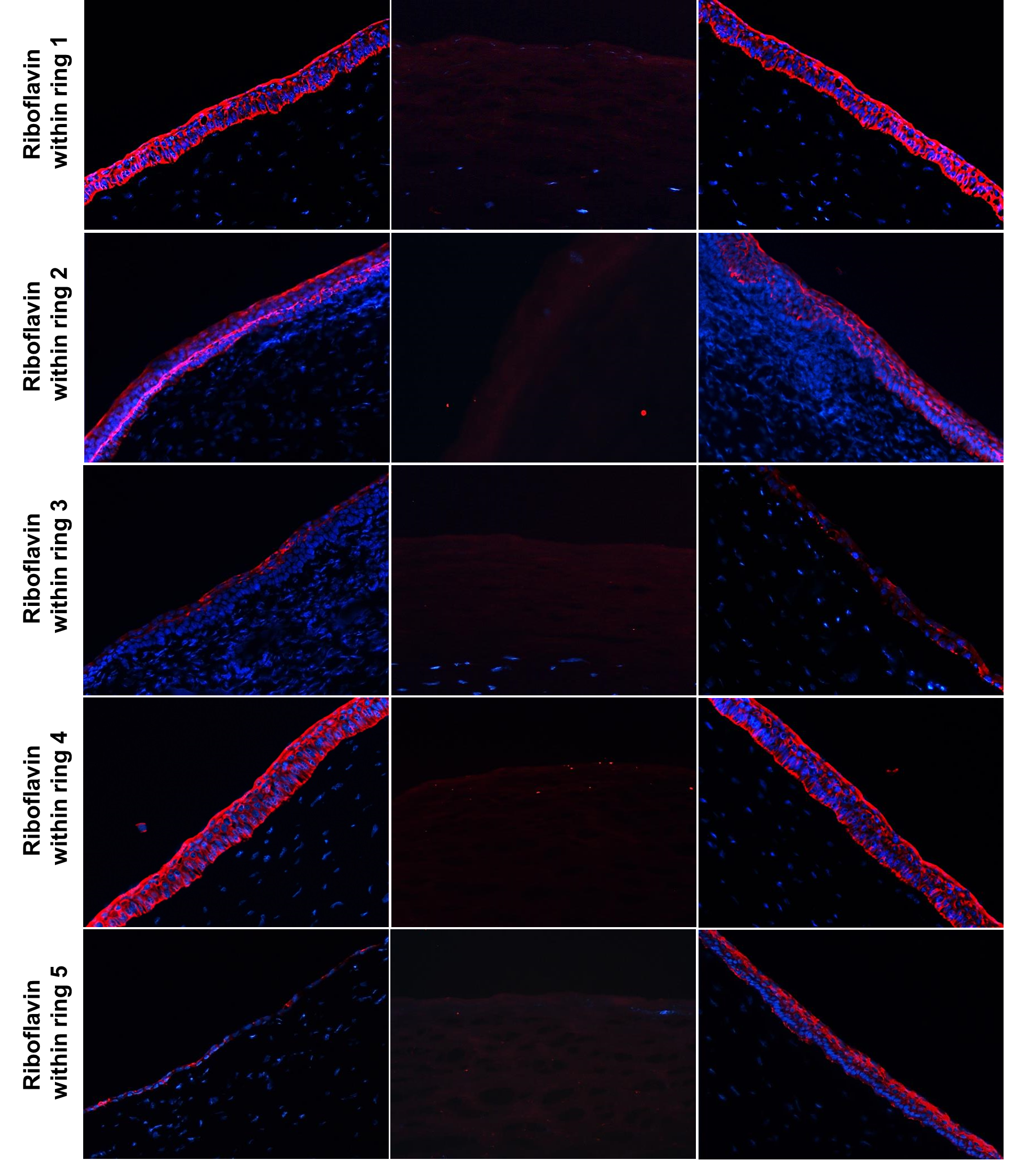

Supplement: Supplementary 3 — Figure 3: CK3/12 immunostaining in the corneas treated with riboflavin using a ring, one day after CXL. [file 6854298.f3.tif]
